# Detraining Effects on Musculoskeletal Parameters in Early Postmenopausal Osteopenic Women: 3-Month Follow-Up of the Randomized Controlled ACTLIFE Study

**DOI:** 10.1007/s00223-021-00829-0

**Published:** 2021-03-12

**Authors:** Wolfgang Kemmler, Michael Hettchen, Matthias Kohl, Marie Murphy, Laura Bragonzoni, Mikko Julin, Tapani Risto, Simon von Stengel

**Affiliations:** 1 Institute of Medical Physics, Friedrich-Alexander University Erlangen-Nürnberg (FAU), Henkestrasse 91, 91052 Erlangen, Germany; 2 Department of Medical and Life Sciences, University of Furtwangen, Schwenningen, Germany; 3 Doctoral College, Ulster University, Newtownabbey, Co. Antrim, Northern Ireland, UK; 4 University of Bologna, Campus Rimini, Rimini, Italy; 5 Laurea University of Applied Sciences, Espoo, Finland

**Keywords:** Detraining, High-intensity exercise, Bone mineral density, Lean body mass, Strength and power

## Abstract

Periods of absence from supervised group exercise while maintaining physical activity might be a frequent pattern in adults' exercise habits. The aim of the present study was to determine detraining effects on musculoskeletal outcomes after a 3-month detraining period in early post-menopausal, osteopenic women. Due to the COVID-19 pandemic, we terminated the 18-month randomized controlled ACTLIFE exercise intervention immediately after the 13-month follow-up assessment. This put an abrupt stop to the high-intensity aerobic and resistance group exercise sessions undertaken three times per week by the exercise group (EG: *n* = 27) and the gentle exercise program performed once per week for the attention control group (CG: *n* = 27); but both groups were permitted to conduct individual outdoor activity for the 3-month lock-down period. Study endpoints were lean body mass (LBM), bone mineral density (BMD) at the lumbar spine (LS), maximum hip-/leg extension strength and power. Detraining-induced reductions of LBM, hip/leg strength and power (but not BMD-LS) were significantly greater (*p* < 0.001 to *p* = 0.044) compared with the CG. Significant exercise effects, i.e. differences between EG and CG, present after 13 months of exercise, were lost after 3 months of detraining for LBM (*p* = 0.157) and BMD-LS (*p* = 0.065), but not for strength (*p* < 0.001) and power (*p* < 0.001). Of note, self-reported individual outdoor activities and exercise increased by about 40% in both groups during the lock-down period. Three months' absence from a supervised group exercise protocol resulted in considerable detraining effects for musculoskeletal parameters. Thus, exercise programs for adults should be continuous rather than intermittent.

Trial registration number: ClinicalTrials.gov: NCT04420806, 06.05.2020.

## Introduction

Regular exercise is thought to mitigate negative consequences of the menopausal transition which considerably affect women’s lives. In the ACTLIFE study [1, 2] canceled prematurely due to COVID-19, we demonstrated significant positive effects of a 13-month supervised exercise program on body composition, Bone Mineral Density, menopausal symptoms and physical fitness. The three-month lock down of all training facilities in Bavaria, Germany provided an opportunity to examine the effect of a short break in a structured group exercise programs on these parameters. Due to the large number of health care providers and commercial suppliers who do not provide continuous exercise programs, this issue is highly relevant. In a recent 16-week study, on younger (20–35 years) and older adults (60–75 years), Bickel et al. [3], reported significant reductions of lean body mass after only 8 weeks of detraining. Several studies confirmed the deleterious effects of short-moderate training breaks/detraining after aerobic, resistance or concurrent exercise in non-athletic cohorts. However, most of these studies (a) focus on people 60 years and older (e.g. [4–12], and/or applied (b) short training periods^1^ (e.g. [6, 11, 13, 14] and/or (c) long detraining periods (i.e. 24 weeks and longer, e.g. [10, 15–18]), the latter particularly refer to detraining studies on bone mineral density (BMD) (e.g. [19–21].

We aimed to determine the effect of moderate periods of absence from intense multimodal group exercise (3 months) on musculoskeletal parameters in a cohort of early postmenopausal women.

Our main hypotheses are 3 months of detraining after 13 months of exercise leads to significantly higher reductions of (a) lean body mass (primary hypothesis), (b) BMD at the lumbar spine (LS), (c) maximum hip/leg extension strength and (d) power compared with an attention control group (i.e. differences in detraining changes between the EG and the CG).

We further hypothesize that after 13 months of training and 3 months of detraining, the significant training effects for the parameters (a–d) are lost (i.e. difference in overall changes from baseline to 16 month follow-up between the EG vs. the CG.)

## Methods

The present work is part of the ACTLIFE project, a European Project that focuses on the development and dissemination of best practice exercise protocols for therapy and prevention of osteoporosis. ACTLIFE was designed as an 18-month multimodal randomized control exercise trial that addressed the menopausal risk factor with specific regard to musculoskeletal parameters. However, due to the COVID-19-induced lockdown of all training facilities as of March 17th 2020, ACTLIFE had to stop immediately after the 13-month follow-up assessment. After approval from the FAU Ethics Committee (number 118_18b), informed consent of all study participants and study registration (ClinicalTrials.gov: NCT04420806), the 3-month (detraining) follow-up assessment was conducted in mid-June 2020, one week after the reopening of assessment and training facilities (June 8th, 2020).

### Participants

**Fig. 1:**
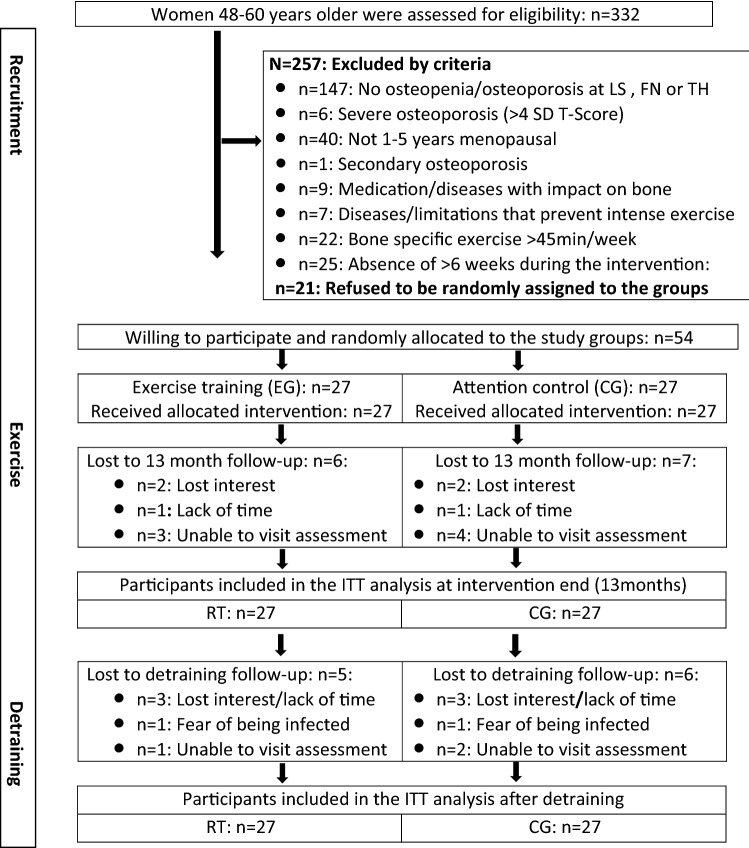
Participant flow through the ACTLIFE study

The recruitment process of the ACTLIFE-RCT has already been reported in detail. In summary, women were eligible if they met the following criteria: (a) 48–60 years old, (b) early-menopause status (1–5 years of amenorrhea), (c) osteopenia or osteoporosis at the LS, femoral neck (FN) or total hip (TH), (d) no medication, conditions and diseases known to affect bone metabolism or contraindicate group exercise or tests, (e) no high impact or resistance exercise in the past 5 years, (f) no secondary osteoporosis or osteoporotic fractures, (g) no acute or recent history of cancer (last 5 years), (h) alcohol consumption < 60 g/d on 5 days/week) (Fig. 1). Applying these criteria 54 women eligible and willing to participate were randomly assigned to the two study groups (Fig. 1).

### Randomization Procedures

Briefly, participants stratified for LS-BMD were randomly allocated to exercise (*n* = 27) and control group (*n* = 27) by drawing lots. Neither researchers nor participants knew the allocation beforehand (“allocation concealment”).

### Blinding

The blinding strategy of ACTLIFE included outcome assessors and test assistants who were unaware of and not allowed to ask the participants' group assignment (EG or CG).

### Study Procedure

Apart from the high-intensity multimodal exercise protocol for the EG and the low intensity/low volume program for the CG, all participants were provided with cholecalciferol (Vit-D) and calcium (Ca) supplements according to recent recommendations (i.e. 800 IU/day Vit-D; 1000 mg/day Ca [22], details see below). In contrast to the exercise protocol that had to be stopped in mid-March 2020, calcium and Vit-D was provided up to study end in mid-June 2020. Apart from the interventions, participants were requested to maintain their usual dietary intake and lifestyle including habitual physical activity and exercise habits.

### Intervention

#### Exercise Intervention

All EG and CG participants started the intervention in Mid-February 2019 and had to stop exercising in Mid-March 2020 due to the corona-induced lockdown of all training facilities in Bavaria, Germany.

#### Exercise Group

The EG exercise protocol has been described in detail previously [1, 2]; thus we only outline it here. In summary, our exercise protocol can be considered as a periodized high-intensity approach that focused on musculoskeletal parameters. In general, we structured our exercise protocol into 8–12-week blocks of high-intensity/high-effort exercise, interspersed by 4–5 weeks of lower exercise intensity/volume. Applying consistently 3 sessions/week we realized 15–20 min of aerobic dance in a high-intensity interval training (HIIT) mode^2^ with moderate to high ground reaction forces (GRF),^3^ jumping^4^ and periodized high intensity (60–85% 1 RM)/high effort^5^ resistance training (HIT-RT)[24]. The two sessions conducted in our lab focused on HIIT and HIT-RT, the latter in a single set circuit mode using barbells/body weight. The third session was conducted in a multiple set mode on resistance exercise machines in a dedicated gym. Independently of the setting, RT-exercises (10–14 exercises) addressed all the main muscle groups. Apart from high intensity/effort, we manipulated movement velocity during RT by applying periods of explosive movements in the concentric phase. The consistently supervised 8–12-week phases were structured into 2–3 linearly periodized 4-week periods with each 4th week as a regeneration week with lower intensity (60–65% 1 RM) and lower effort (non-repetition maximum). During the 4–5-week intermitted periods, one circuit session (60–75% 1 RM, however not to RM [23]), one 45 min session of stretching and easy floor exercises (see control group) and one 15 min video-guided home training session (see control group) were scheduled.

### Control Group

Exercise in the attention control group focused on stability, flexibility and well-being, albeit with strong emphasis on applying an exercise protocol unlikely to affect “bone”, “body composition” or “maximum strength/power”. During the 13-month intervention period, we completed two cycles of 12 weeks with one session of 45 min/week of consistently supervised group exercises intermitted by 12 and 14 weeks of non-supervised, video-guided home exercise (15 min).

### Vitamin-D and Calcium Supplementation

All participants were provided with 5000 IE/week of cholecalciferol (MYPROTEIN, Cheshire, UK) [22], independently of their baseline 25OH D levels (Table 1). Based on dietary calcium intake (“calcium questionnaire”, Rheumaliga, Switzerland), we supplemented calcium carbonate capsules (Sankt Bernhard, Bad Dietzenbach, Germany) to realize a calcium intake of 1000 mg/day [22].

**Table 1:** Baseline and 13-month follow-up characteristics of the ACTLIFE-RCT study

Variable	CG (*n* = 27) MV ± SD		EG (n = 27) MV ± SD	
	Baseline	13 months	Baseline	13 months
Age [years]	54.5 ± 1.6	55.6 ± 1.6	53.6 ± 2.0	54.6 ± 2.0
Body height [cm]	164.5 ± 8.2	164.5 ± 8.2	164.2 ± 6.0	164.2 ± 6
Body mass [kg]	67.4 ± 14.6	68.4 ± 14.1	64.0 ± 9.6	63.7 ± 10.2
Calcium intake [mg/day]	642 ± 265	671 ± 301	645 ± 252	666 ± 274
Vit-D level (25-OHD) [ng/ml]	21.6 ± 10.8	30.7 ± 11.6	27.8 ± 11.7	37.1 ± 12.8
Years after menopause [year]	3.5 ± 1.1	4.5 ± 1.1	3.7 ± 1.0	4.8 ± 1.0
Exercise volume [min/week]	46 ± 38	51 ± 45	64 ± 48	57 ± 42
Individual outdoor activity [min/week]	110 ± 74	119 ± 90	131 ± 94	126 ± 88
Waist circumference [cm]	91.1 ± 9.9	90.8 ± 9.7	87.8 ± 8.6	86.0 ± 8.7
Energy intake^a^ [kcal/day]	2067 ± 355	2088 ± 387	2009 ± 444	2051 ± 403
Protein intake [g/kg/body mass/day]	1.20 ± 0.21	1.16 ± 0.19	1.18 ± 0.27	1.21 ± 0.23
Ovariectomy < 50 years [*n*]	0	–	1^b^	–
Family disposition^c^ [*n*]	9	–	7	–

^a^As determined by a 4-day dietary protocol, see methods

^b^At age 47 years

^c^Fragility fractures or verified osteoporosis in close relatives (parents, aunts, uncles, grandparents)

### Compliance with the Exercise Intervention

Instructors and chip card systems were used to monitor participants' exercise attendance. Adherence to the exercise protocol, particularly for exercise intensity, was checked (1) by the instructors monitoring the load/repetition proportion during the sessions and (when necessary) asking participants to work with more effort and (2) by reviewing the participants' training logs after the 8–12-week meso-cycles.

### Study Outcomes

#### Primary Study Outcome(s)

- Changes of (soft) lean body mass determined by dual-energy X-ray absorptiometry (DXA) from intervention end (13 months) to 3-month detraining follow-up.

#### Secondary Study Outcomes

- Changes of BMD at the lumbar spine as determined by DXA from intervention end (13 months) to 3-month detraining follow-up.
- Maximum dynamic hip-/leg-extension strength changes as determined by an isokinetic leg press from intervention end (13 months) to 3-month detraining follow-up.
- Maximum dynamic hip-/leg-extension power (“jumping height”) as determined by a force plate from intervention end (13 months) to 3-month detraining follow-up.

### Changes of Trial Outcomes After Trial Commencement

Due to the rapid and strict COVID-19 lockdown, we were unable to repeat the MRI assessment at intervention end (13 months) and correspondingly did not apply MRI after the detraining period (i.e. 3-month follow-up).

### Assessments

Standardized testing and assessments were used at each timepoint. Participants were briefed and asked to avoid changes in dietary intake or high physical activity 48 h prior to the tests. All the tests were conducted at about the same time of day (± 90 min), using the same calibrated devices and/or specifications and the same protocols, but not always (i.e. strength/power assessments) by the same test assistant.

Body height was determined using a Holtain stadiometer (Crymych Dyfed., Great Britain). Body mass was assessed using direct-segmental, multi-frequency Bio-Impedance-Analysis (DSM-BIA; InBody 770, Seoul, Korea), which was also used as a backup assessment for body composition (results not reported here). Areal BMD and body composition were assessed by DXA (QDR 4500a, Discovery-upgrade, Hologic Inc., Bedford, USA). Regions of interest on BMD and regional body composition was segmented using the “compare mode”, so that area and placement of the baseline assessment were reproduced exactly during all the FU assessments.

Maximum isokinetic leg-/hip-extensor strength was assessed with an isokinetic leg press (CON-TREX LP, Physiomed, Laipersdorf, Germany). The range of motion was 30°–90° within the knee angle; velocity of the movement was 0.2 m/s. After familiarization with the testing procedure five reps with maximum effort (“push as strongly as possible”) were conducted.

Lower extremity power was determined by a countermovement jump (CMJ) with hands on hips (i.e. no arm swing). Participants were asked to “jump as high as possible” starting from an upright position. Participants were requested to maintain extension in the hip, knee, and ankle joints after take-off to prevent any additional flight time by flexing their legs during landing. Tests were conducted on a force platform (KMP Newton GmbH, Stein, Germany). The jumping height was calculated automatically by the software provided by the manufacturer based on ground reaction forces.

A standardized baseline questionnaire [25], a 4-day dietary protocol (Freiburger Ernährungsprotokoll, see below) and specific physical activity and exercise questionnaire [26–28] asked for (a) demographic parameters; (b) diseases, physical limitations and pharmacologic therapy under special consideration of osteoporosis risk and ability to frequently conduct intensive exercise; (c) dietary supplements; (d) pain frequency and severity at the lumbar spine region [29] and (e) lifestyle, including physical activity and exercise [26–28].

During follow-up (FU) the same questionnaires aimed to evaluate changes from baseline, additionally we also asked for changes of lifestyle, diet, exercise, and pain levels in the FU questionnaire. This questionnaire focused mainly on changes in pharmacologic therapy, diseases or operations i.e. also parameters that might have affected the present study outcomes. Of key importance for reliable results, we placed strong emphasis on consistency, completeness and accuracy by checking the completed questionnaires together with the participants.

Dietary intake was recorded on 3 weekdays and one weekend day characteristic for dietary habits at baseline and after 7 months, 13 months and 16 months. Participants were provided with simple diet records (Freiburger Nutrition Record, nutri-science, Hausach, Germany) which were analyzed consistently by the same research assistant. In cases of unlikely results, (e.g. energy intake < 1000 kcal/day or > 3500 kcal/day), the women were requested to provide another dietary record based on more representative days.

### Sample Size Calculation

The initial sample size calculation was based on “BMD changes at the LS” after 18 months. In order to generate an estimated effect (Δ-EG vs Δ-CG) on BMD-LS of 2.0 ± 2.5% [30, 31], the sample size required to generate 80% power (1 − β) and alpha = 0.05 was 25 participants per group. We included 27 participants to allow for drop-outs within an additional per protocol analysis for the primary study outcome.

### Statistical Analysis

We conducted an intention to treat (ITT) analysis that included all participants initially assigned to the EG and CG. ITT was performed using R statistics software [32], in combination with Amelia II [33]. The full data set was used for multiple imputations. Imputation was repeated 100 times. According to imputation diagnostic plots, imputation worked well. In addition to the ITT analysis, we conducted a per protocol analysis for the primary hypothesis, that included all participants with 13- and 16-month data, independently of their compliance. Normal distribution of the study endpoints was checked by statistical (Shapiro–Wilks) and graphical (qq-plots) procedures. In order to compare changes for the detraining period between the EG and the CG (primary hypothesis) we applied an ANCOVA adjusted for 13-month data. In parallel, to determine overall effects after training and detraining (secondary hypothesis), we also applied an ANCOVA that adjusted on baseline data. Finally the changes over time inside the groups i.e. training (baseline to 3 months) and detraining effects (13–16 months) were investigated by paired *t* tests applying the approach of Barnard and Rubin [34]. Two-tailed tests were applied and significance was accepted at *p* < 0.05.

## Results

Participants’ baseline and 13-month characteristics are displayed in Table 1. Albeit non-significant, 25-OHD levels vary considerably, while other participant characteristics at baseline and intervention end (13 months) were similarly distributed.

In summary, five women in the EG and six women of the CG were lost to 16-month follow-up (Fig. 1). Altogether six participants quit during the intervention period. With respect to the 3-month detraining follow-up, two participants refused to be assessed due to fear of being infected, two other women were unable to visit the assessments (Fig. 1).

Attendance rate was 79 ± 12% in the EG and 78 ± 14% in the CG. The monitoring of “effort” by checking the relationship of reps and load selected to realize RM specification indicates that in one fourth to one third of the cases women did not follow our RM prescription. This refers particularly to the first 12-week mesocycle.

### Primary and Secondary Study Outcomes

**Fig. 2:**
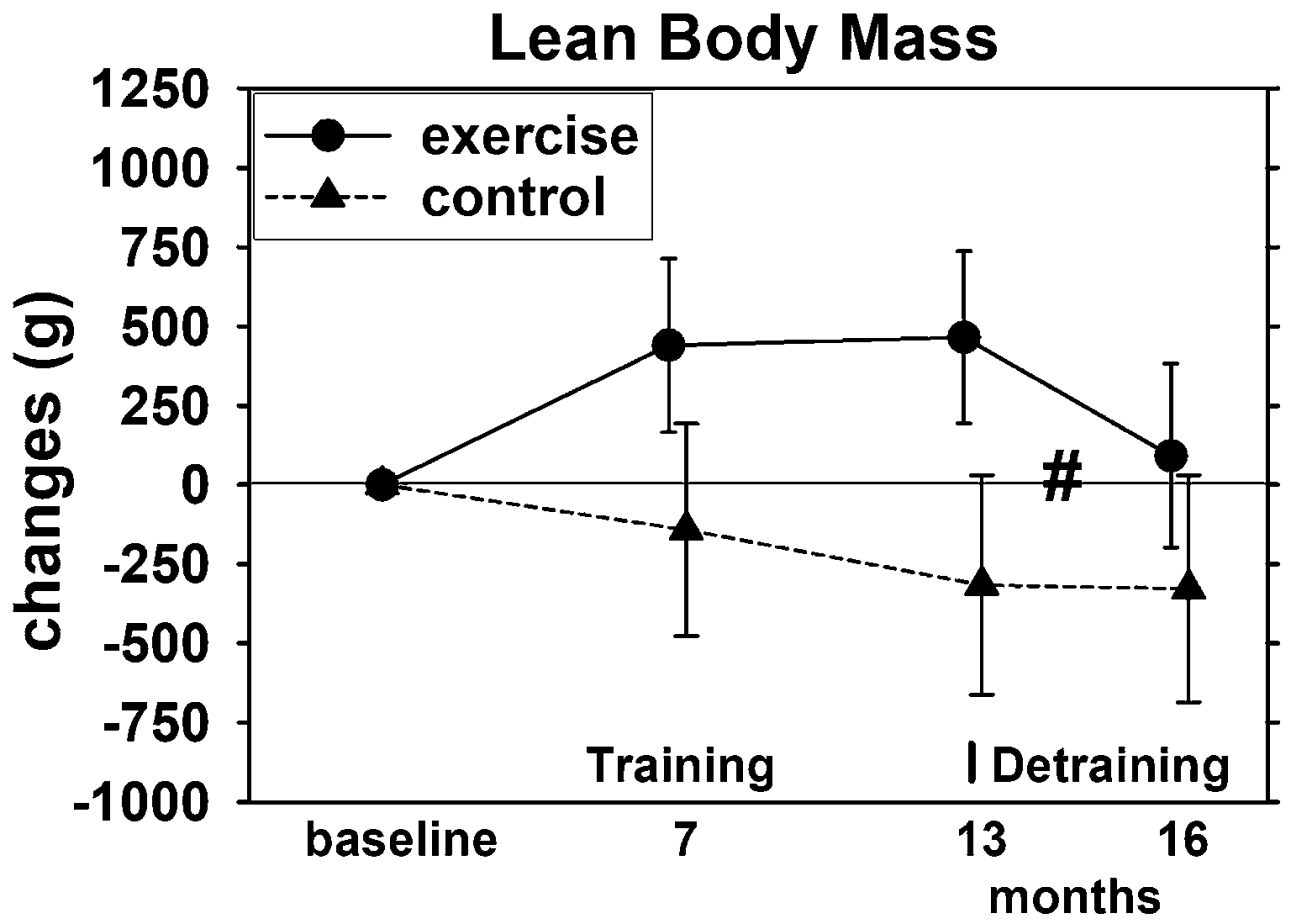
Mean values and 95% CI for changes of Lean Body Mass (LBM) after training and detraining based on the intention to treat analysis (*n* = 27 in the EG and CG). # significant difference in changes from 13-month (end of intervention) to 3-month FU (16 month) in the EG vs. CG

During the intervention period, LBM increased significantly in the EG (*p* < 0.001) and decreased non-significantly (*p* = 0.106) in the CG (Fig. 2), resulting in significant (*p* < 0.001) exercise effects after 13 months of exercise. However, with respect to the primary hypothesis, LBM reduction during the detraining phase was significant for the EG (*p* = 0.019), while no relevant changes were observed for the CG. Most importantly, however, we found significant differences (*p* = 0.044) in LBM changes (study end—3 month FU) between the EG and the CG (primary hypothesis). Results of the ITT-analysis with respect to the primary hypothesis were supported by the per protocol analysis. Moreover, after 16 months, no more significant exercise effects (baseline to 16 months) on LBM were observed (*p* = 0.157) (secondary hypothesis).

Accordingly, we accepted the main hypothesis (a) that 3 months of detraining after 13 months of exercise lead to significant higher reductions of lean body mass compared to an attention control group and (subordinate hypothesis) that exercise effects on LBM observed at intervention end (13 months) were lost after 3 months of detraining (16 month).

**Fig. 3:**
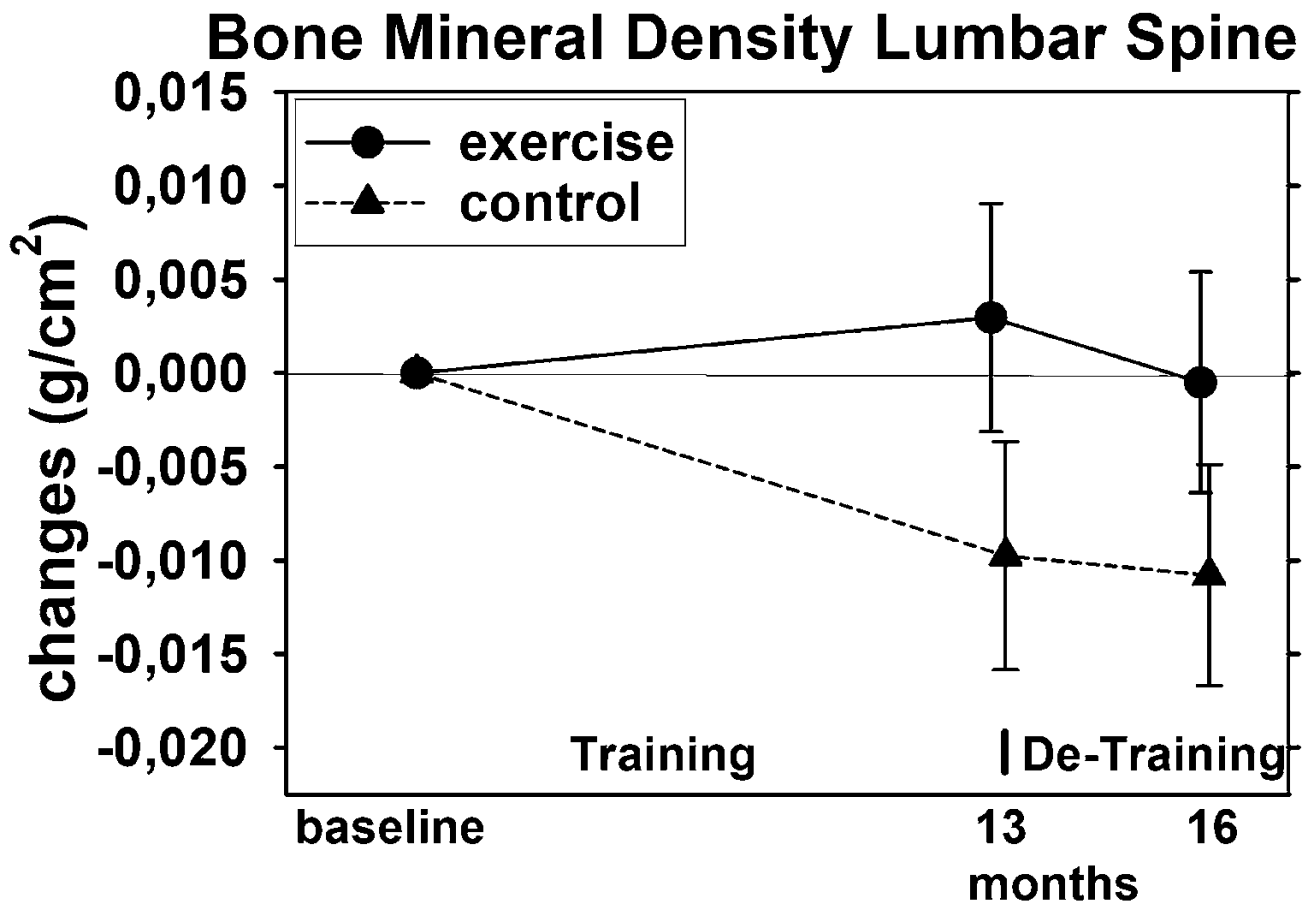
Mean values and 95% CI for changes of BMD at the lumbar spine after training and detraining based on the intention to treat analysis (*n* = 27 in the EG and CG)

After 13 months of intervention, significant BMD reductions were determined for the CG (*p* = 0.004) and slight increases were observed in the EG (*p* = 0.337). In summary, exercise effects (EG vs. CG) after 13 months were significant (*p* = 0.027). However, more importantly three months of detraining reduced LS-BMD in the EG slightly below baseline values although the reduction was not significant (*p* = 0.115), while no relevant negative BMD change during the detraining phase was observed for the CG. Nevertheless, changes during the detraining phase did not differ significantly between the two groups (*p* = 0.523). This result is confirmed by the per protocol analysis (*p* = 0.477). Moreover, the overall effect after 16 months of training and detraining was non-significant (*p* = 0.065) (Fig. 3).

With respect to BMD at the LS, we revised our hypothesis (b) that 3 months of detraining after 13 months of exercise leads to significant higher reductions of BMD-LS compared to an attention control group; however, we confirmed that exercise effects for BMD observed at intervention end (13 months) were lost after 3 months of detraining.

**Fig. 4:**
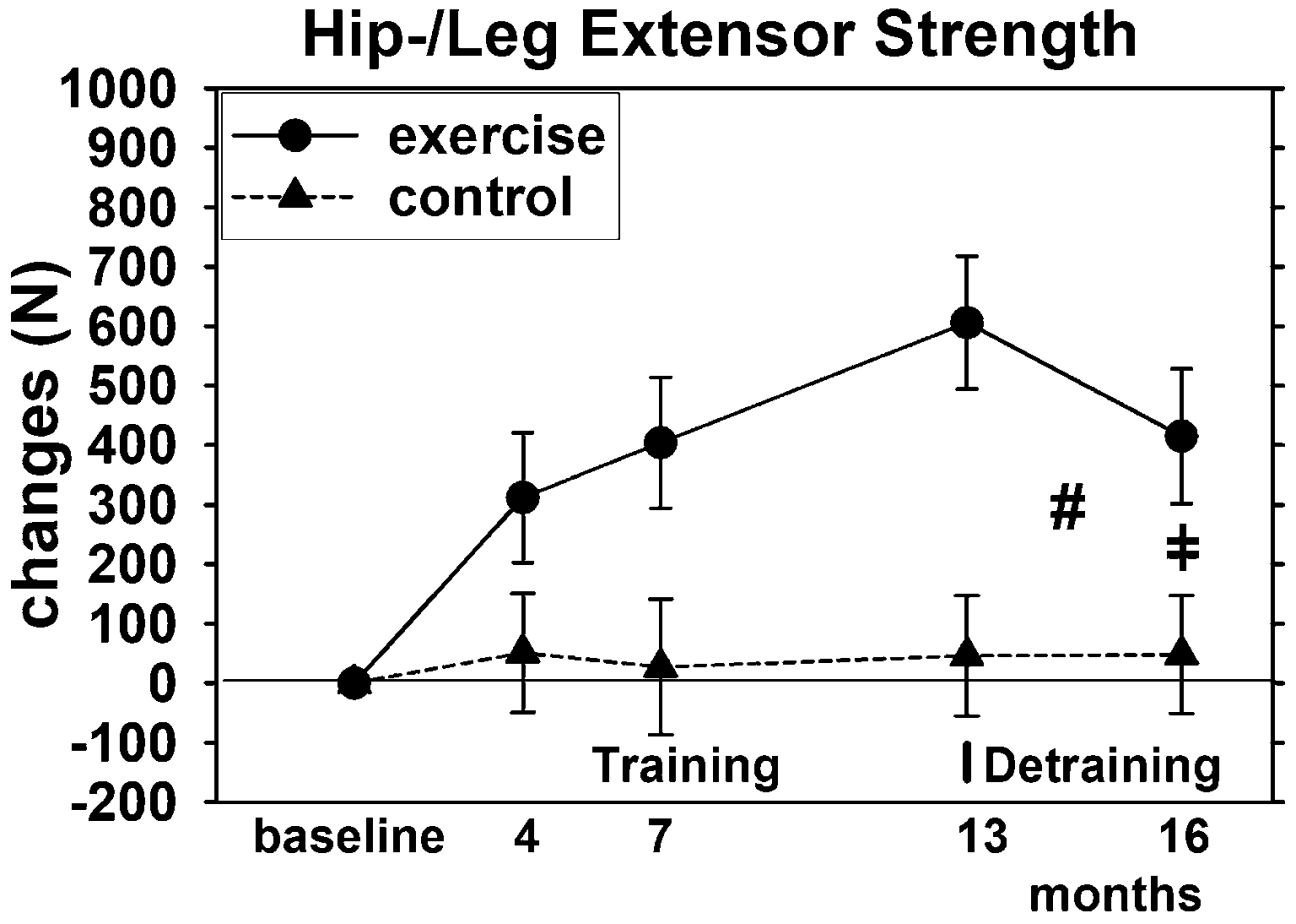
Mean values and 95% CI for changes of hip-leg extensor strength after training and detraining based on the intention to treat analysis (*n* = 27 in the EG and CG). # significant difference in changes from 13-month (end of intervention) to 3-month FU (16 month) in the EG vs. CG. ǂ significant different changes from baseline to 16 month (i.e. “overall effects”)

**Fig. 5:**
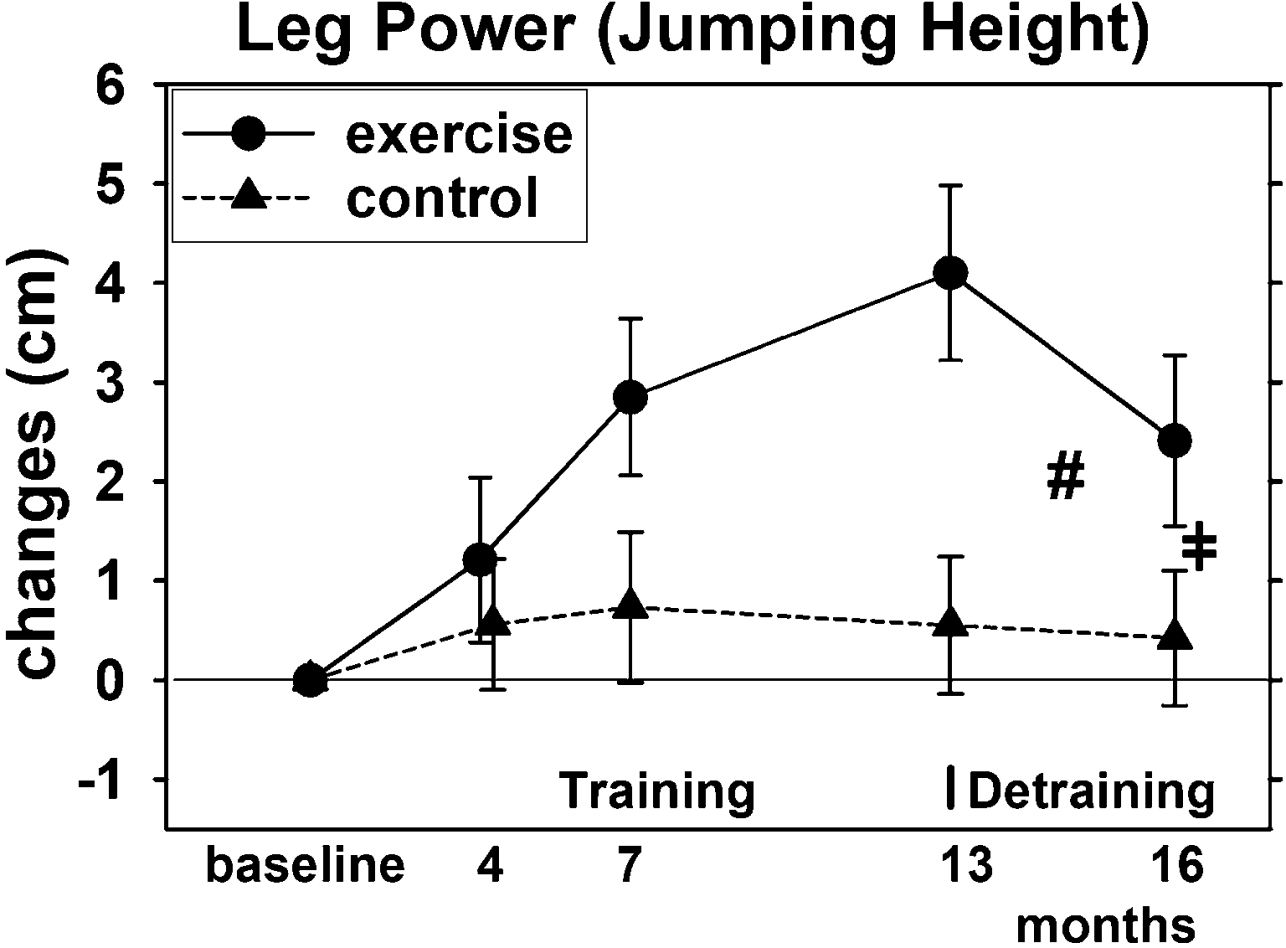
Mean values and 95% CI for changes of leg power after training and detraining based on the intention to treat analysis (*n* = 27 in the EG and CG). # significant difference in changes from 13-month (end of intervention) to 3-month FU (16 month) in the EG vs. CG. ǂ significant different changes from baseline to 16 month (i.e. “overall effects”)

Maximum hip-/leg-extension strength and power of the EG increased significantly during the intervention phase (both *p* = 0.001) and increased slightly (significant for 7-month FU) in the CG (Figs. 4 and 5) resulting in significant (*p* < 0.001) exercise effects after 13 months of exercise.

Strength and power reductions during the detraining phase were significant for the EG (both *p* < 0.0.001), and differ significantly (both *p* < 0.001) from changes in the CG. The latter result was confirmed by the additional per protocol analysis. After 16 months, i.e. 13 months of training and 3 months of detraining, the exercise effect (between group difference) was still significant (both *p* < 0.001) (Figs. 4 and 5).

Thus, we confirmed hypotheses (c) and (d) that 3 months of detraining lead to significant higher reductions of maximum hip-/leg-extension strength and power compared to an attention control group. However, we revised our hypotheses that significant exercise effects were lost during the detraining period.

### Confounding Parameters

During the detraining period no relevant changes or between group differences for dietary intake parameters (i.e. energy, carbohydrate, fat, protein, alcohol intake), pharmacologic therapy or diseases were observed. Habitual physical activity was maintained in both groups, however, as determined by a dedicated questionnaire, the volume of individual aerobic outdoor activities (i.e. brisk walking, cycling, jogging) increased significantly (*p* < 0.001) in both groups by 41% (EG) and 37% (CG). In contrast, although most women worked from home (“home office”) during the lockdown, relevant changes of occupational physical activity were not reported. Of further interest, all but four participants of the EG and CG each conducted the 15 min exercise video at least once per week (EG: 1.4 ± 0.7 vs. CG: 1.7 ± 1.0 sessions/week).

## Discussion

In this study, we clearly confirmed the deleterious effect of short-moderate term detraining periods on musculoskeletal parameters and menopausal complaints in early postmenopausal women. Of note, participants were not physically inactive during the ACTLIFE detraining period, but significantly increased individual aerobic outdoor activities (e.g. walking) during the three months. Although we cannot determine whether this affected our results, we speculate that together with the continued application of the 15 min video-guided home training session, it might have attenuated the negative effect of detraining on our outcomes.

Reviewing our results in detail, the significant reduction of LBM to baseline values in only 3 months of absence of high-intensity aerobic and resistance exercise is particularly remarkable (Fig. 2). In one of the few comparable studies on combined aerobic and RT exercise (AS), Douda et al. [7] applied four 9-month exercise blocks followed by 3 months of detraining each. The authors reported regular (non-significant) decreases of LBM after the training phase that consistently reach pre-training values in women 60 years + . However, apart from one 9-month block, the AS-protocol did not significantly increase LBM during the training phases. In another, albeit much shorter, trial (16 weeks), Bickel et al. [3] applied an intense multiple set RT for the lower limbs in older adults (60–75 years)^6^ and reported significant reductions in thigh lean mass that dropped below baseline values after only 8 weeks of detraining. Of note, one RM knee extension (KE) strength did not decrease significantly with 6 months detraining (55.6 to 50.0 kg), and still significantly exceeded baseline values (40.8 kg). Although in parallel to LBM, maximum hip-leg strength decreased significantly in the EG of the present study and detraining changes differ significantly from the CG (*p* < 0.001), the overall effect (EG vs. CG) is still significant after 16 months of observation and maximum strength in the EG still significantly exceeded (*p* < 0.001) baseline. This might be attributed to the aspect that neuromuscular effects which contribute to strength and power are more resistant to detraining compared with hypertrophic effects [35, 36]. Consequently, this will result in higher preservation of strength/power compared to muscle mass gains during detraining (e.g. [3, 5, 12, 37, 38]). However, these data are not undisputed. In their 8-week and 16-week RT-studies on older people (70–80 years and 90 ± 1 years, respectively), Lovell et al. [39] and Fiatarone et al. [40] observed significant reductions in maximum leg strength (1 RM squat) after only 4 weeks of detraining—nevertheless, detraining 1 RM still differs significantly from baseline. On the other hand, a 24-week RT-protocol with middle aged (37–44 years.) and older adults (63–78 years), [35], did not show reductions of 1 RM and other neuromuscular parameters of leg extension strength and power^7^ after a 3-week detraining phase. Therefore, apart from the length of the detraining phase and of course from training effects,^8^ there is some evidence that stability of adaption increases with the duration over which training is performed [41]. Reviewing studies with longer intervention periods, after 9 months of multicomponent exercise and 3 months of detraining, Esain et al. [8] reported maintained upper and lower limb strength and endurance in his cohort of adults 65 years and older. This result was confirmed by Vuori et al. [42], who did not report relevant changes of maximum leg extensor strength after 3 months of detraining following a 12-month RT with young women (19–27 years). In contrast, Carvalo et al. [4], who applied a 8-month multicomponent exercise program, listed significant reductions of functional strength parameters^9^ after 3 months of detraining in older women (64–85 years.), although leg extension but not arm flexor strength still significantly exceeded baseline values.

Due to the need for longer training (…and detraining?) periods due to slower bone metabolism [43] and the more discreet training effects of training on bone (compared to muscle mass or strength) [44], detraining studies on BMD are rare [19–21, 42, 45, 46]. Two studies that applied similar long training (8 months and 12 months) and detraining periods (3 months and 4 months) [42, 46], albeit in young women (18–27 years.), confirmed our results. Both authors reported moderate, non-significant decreases (≈ 1.5%) of BMD at the LS after an exercise-induced gain of 2.0% [42] to 3–3.5% [46]. Another study with older osteosarcopenic men who also underwent a corona-induced training break after 18 months of high-intensity dynamic resistance exercise [45], reported a significant reduction of BMD-LS, muscle mass and function after 6 months of detraining. As with the present study, training-induced effects after the detraining period remained significant for muscle mass and function, but not for BMD of the LS.

In summary, it is difficult to estimate the optimum duration of a training break that might allow full regeneration/resensitization of the given musculoskeletal parameter (i.e. strength, muscle, bone), without resulting in unintended detraining effects. With respect to bone, Saxon et al. [47] reported the most favorable effect on bone strength for intermitted rest protocols with 5 week of “time off”. Conversely, regular bone resensitization cycles of 5–6 weeks after 10–12 week phases of high intensity/effort/velocity as applied in this and other studies [48–50] of ours, might prevent optimum development of muscle mass [3] and/or strength [39, 51].^10^ However, one has to bear in mind that in contrast to the detraining approaches cited above, we did not stop the exercise protocol abruptly but only reduced exercise intensity, type and volume of high impact and RT-exercise to a lower level during our “regeneration phases”. In this context, data provided by Bickel et al. [3] indicate that reductions to one third of the initial RT exercise dose^11^ maintain exercise gains in (thigh) muscle-mass and particular strength for 8 weeks and longer.

Our study has several limitations. (1) First of all, this was not a preplanned detraining study. After 4 weeks of detraining we decided to discontinue the study and focus on the present detraining issue. However, the COVID-19 lockdown of all training facilities ensured that none of the women continued the relevant components of the exercise protocol of the present study. (2) On the other hand, since the Bavarian COVID-19 regulation allowed individual outdoor activities, the (self-reported) volume of aerobic exercise as determined by questionnaire increased significantly in both groups. Further, most participants of the EG and CG reported that they conducted the 15 min exercise video at least once per week. (3) All the women were supplemented with recommended doses of cholecalciferol and calcium [22] throughout the detraining period. This increases the evidence^12^ and our confidence that any reductions in musculoskeletal parameters were predominately related to detraining effects. (4) Also due to the unplanned implementation of detraining, we did not conduct a sample size analysis for detraining and so it might be underpowered for adequately addressing some of the endpoints. (5) We focused on study endpoints that demonstrated significant effects during the intervention period. Consequently, we did not include BMD at the total hip, for example, which failed to demonstrate significant positive effects (*p* = 0.129) after 13 months of exercise. (6) Due to the rapid and consequent lock-down and the still prevalent pandemic, we lost a considerable number of participants to 13-month and 16-month follow-up (Fig. 1). We applied ITT with multiple imputation; however, even the most sophisticated imputation approach does not completely reflect reality. This situation led us to conduct an additional per protocol analysis that included only participants with follow-up data.

In conclusion, despite increases in aerobic outdoor activities and home exercise, 3 months of absence from a supervised high-intensity group exercise protocol resulted in detraining effects that were significant for lean body mass, muscle strength and power. Thus, although it might be not completely legitimate to generalize our results to other age groups [3, 52], roughly in line with the present detraining literature, we confirmed the general need for continuous rather than intermitted exercise programs [7]. This however, does not contradict the application of short regeneration/resensitization phases during periodized exercise protocols.

## Data Availability

The datasets generated and/or analyzed during the current study are available from the corresponding author on reasonable request.

## Footnotes

1 …8–12 weeks, i.e. a period that might be too short to determine the full amount of adaptation to exercise.

2 20 min of aerobic dance; after 5 min of warm up either 10 × 60 s at  85% HFmax interspersed by 60 s of lower exercise intensity or a corresponding 30 s/30 s protocol was applied.

3 Up to 120 movements/session with GRF:  2.0–3.0 × body mass.

4 Up to 50–60 bilateral jumps/session with GRF up to 4–4.5 × body weight.

5 Applying “work to repetition maximum” i.e. set endpoint when trainees complete the final repetition possible whereby if the next repetition were attempted they would definitely achieve muscular failure. [23] and intensifying strategies (i.e. supersets, drop-sets) [24]

6 The authors [3] observed significant increases of TLM (4.4%) and KE strength (36%) after 16 weeks of training.

7 apart from a significant decrease of maximum isometric force (6%) during unilateral leg extension in the younger subgroup.

8 Detraining effects can only occur after prior training effects; correspondingly higher training effects at least offer a higher potential for detraining effects [39, 40].

9 Chair stand, arm curl [4].

10 The latter review focuses on younger cohorts, however.

11 The authors [3] simply reduced the weekly exercise frequency from three to one session per week. Of note, an exercise volume reduction to one ninth of the initial dose preserved muscle hypertrophy in the young but not old, while strength gains were maintained in both groups.

12 Apart from the observation that no relevant changes of diseases or medication with impact on muscle or bone metabolism were determined during the detraining period.
